# Beyond discrimination: calibration and decision utility are required before GREAT can guide individual ECT decisions

**DOI:** 10.1192/bjo.2026.12048

**Published:** 2026-07-09

**Authors:** Juan Carlos Munguia, Yolly Molina, Patricia Soriano, Isaac Zablah

**Affiliations:** Faculty of Medical Sciences, National Autonomous University of Honduras, Honduras; Directorate of Forensic Medicine, Public Ministry, Tegucigalpa, Honduras

**Keywords:** Electroconvulsive therapy, depression, prediction model, calibration, decision curve analysis

## Abstract

Belz and colleagues present GREAT, a seven-item clinical instrument for predicting electroconvulsive therapy response in unipolar depression, with promising discriminatory validity (AUC 0.841). We identify three methodological gaps – absent calibration, limited sample representativeness and unquantified incremental value – that must be addressed before GREAT can guide individual clinical decisions.

**Figure:**
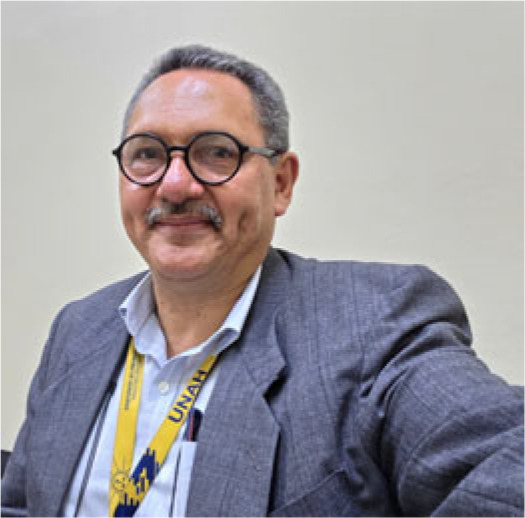

Electroconvulsive therapy (ECT) remains the most effective intervention for severe or treatment-resistant unipolar depression, yet the persistent challenge of anticipating individual response continues to impede timely and equitable treatment allocation. We read with considerable interest the prospective study by Belz and colleagues, who introduce the Göttingen Response to the ECT Assessment Tool (GREAT), a seven-item scoring instrument designed to predict response prior to treatment initiation.^1^ The initiative is scientifically timely: access to ECT remains constrained by resource limitations and institutional barriers in many healthcare systems,^2^ and a validated, infrastructure-independent predictor could meaningfully improve triage efficiency and shared decision-making. GREAT’s pragmatic design, drawing exclusively on clinician-assessed routine data, requiring no neuroimaging or molecular assays and yielding an area under the receiver-operating characteristic curve (AUC) of 0.841, represents a genuine conceptual advance in a field where prior multivariate models have reported comparatively modest performance.^3,4^ We offer three methodological considerations that, if addressed, would substantially strengthen the case for clinical implementation.

## Calibration: the missing half of predictive validity

The most consequential gap in the current analysis concerns calibration, the degree of agreement between predicted and observed response probabilities across the full risk spectrum. The authors present a compelling case for discrimination (the capacity to rank patients by relative response likelihood), but the manuscript is entirely silent on whether the numerical probabilities implied by the GREAT score are accurate at the individual level. These are conceptually distinct properties: a model can achieve a high AUC and still systematically overestimate or underestimate response rates within clinical subgroups.^5,6^ Contemporary prediction-model reporting standards, including TRIPOD + AI guidance, recommend calibration-in-the-large, calibration slope and graphical calibration plots as minimum requirements for any model intended to inform patient-level decisions.^5^ The relevance of this omission is not merely methodological. GREAT’s seven item gradations were established by clinical judgement and are acknowledged by the authors as ‘partly arbitrary’, introducing the possibility of systematic miscalibration at the extremes of the score distribution. Furthermore, 7 patients who exceeded the proposed ≥7-point threshold nonetheless failed to respond, while 2 patients below it achieved clinically meaningful Montgomery-Asberg Depression Rating Scale improvement.^1^ These discordant cases are precisely where calibration determines whether the model’s probabilistic framing supports or misleads bedside reasoning.

## Sample composition and external validity

The preliminary nature of this derivation study warrants explicit attention to sample composition. The cohort is markedly sex-imbalanced, with women representing 80% of included patients (*n* = 36/45) – substantially higher than the female predominance reported in large multicentre ECT registries.^7,8^ The relative predictive weight of age, psychomotor symptoms and pharmacotherapy resistance may differ in male patients, and this imbalance limits generalisability across broader clinical populations. In addition, item-level analyses revealed that four of the seven GREAT items (pharmacotherapy resistance, depression severity, psychotic symptoms and psychomotor symptoms) did not individually achieve statistical significance as predictors of response. In small samples, such non-significant weights can reflect insufficient statistical power rather than true clinical irrelevance, and their inclusion without recalibration may introduce noise into individual predictions.^9^ Two further predictors, childhood trauma and maltreatment, have recently been associated with attenuated ECT outcomes in independent cohorts,^10,11^ as have broader personality disorder traits beyond the borderline subtype.^12^ Incorporating these dimensions into a revised GREAT version and adopting validated staging instruments such as the Maudsley Staging Method for treatment-resistance quantification,^1^ would broaden the instrument’s prognostic scope and clinical relevance.

## Incremental value and the benchmark problem

A central question for any new clinical prediction tool is whether it offers net benefit beyond what clinicians already achieve through structured judgement or parsimonious models. Larger ECT prediction studies employing multivariable regression and Bayesian network approaches on independent, multicentre data-sets have reported substantially more modest performance adjusted *R* ^2^ ≈ 19% and AUC values of 0.686–0.693, even when drawing on broader predictor sets.^3,4^ Machine learning approaches applied to ECT prediction have likewise underperformed on independent samples relative to derivation performance,^13,14^ underscoring that high within-sample accuracy is a necessary but insufficient criterion for clinical recommendation. GREAT’s superior metrics in a single-centre sample of 45 patients may partly reflect optimism inherent to derivation cohorts without external validation, a well-characterised source of inflated performance in clinical prediction research.^9^ To establish GREAT’s added value rigorously, we encourage the authors to compare its performance against a parsimonious baseline model restricted to the two statistically significant item-level predictors identified in their own analysis (age and episode duration), and against structured clinical judgement alone. Decision-curve analysis would then quantify net benefit across clinically plausible threshold probabilities relative to default strategies of treating all eligible or no patients.^15^

In conclusion, these considerations are not intended to diminish the importance of what Belz and colleagues have accomplished. The prospective design, pragmatic item selection and time-efficient administration of GREAT constitute a meaningful step toward individualised ECT planning that respects the resource constraints of real-world clinical environments. We encourage the authors to supplement the current analysis with calibration statistics, to prioritise sex- and age-stratified subgroup reporting, and to embed decision-curve analysis within the planned multicentre validation protocol.^16^ The difficult-to-treat depression framework, which emphasises functional recovery and quality of life beyond binary response thresholds,^17^ may also offer a clinically richer outcome target for the next GREAT iteration. Until calibration and incremental validity are demonstrated prospectively on independent samples, GREAT is best characterised as a promising screening adjunct rather than a standalone decision-support instrument, one whose full potential remains to be realised.

## Data Availability

No new data were generated or analysed for this correspondence.
